# Gemcitabine Plus Nab-Paclitaxel Induces PD-L1 mRNA Expression in Plasma-Derived Microvesicles in Pancreatic Cancer

**DOI:** 10.3390/cancers13153738

**Published:** 2021-07-25

**Authors:** Marzia Del Re, Caterina Vivaldi, Eleonora Rofi, Francesca Salani, Stefania Crucitta, Silvia Catanese, Lorenzo Fontanelli, Valentina Massa, Federico Cucchiara, Lorenzo Fornaro, Annalisa Capuano, Stefano Fogli, Enrico Vasile, Romano Danesi

**Affiliations:** 1 Unit of Clinical Pharmacology and Pharmacogenetics, Department of Clinical and Experimental Medicine, University of Pisa, 56126 Pisa, Italy; marzia.delre@ao-pisa.toscana.it (M.D.R.); eleonora0106@inwind.it (E.R.); s.crucitta@studenti.unipi.it (S.C.); l.fontanelli4@studenti.unipi.it (L.F.); f.cucchiara1@studenti.unipi.it (F.C.); stefano.fogli@unipi.it (S.F.); 2 Department of Translational Research and New Technologies in Medicine, University of Pisa, 56126 Pisa, Italy; caterina.vivaldi@med.unipi.it (C.V.); f.salani1@studenti.unipi.it (F.S.); s.catanese@studenti.unipi.it (S.C.); v.massa@studenti.unipi.it (V.M.); 3 Medical Oncology Unit 2, Azienda Ospedaliero-Universitaria Pisana, 56126 Pisa, Italy; l.fornaro@ao-pisa.toscana.it (L.F.); e.vasile@ao-pisa.toscana.it (E.V.); 4 Department of Experimental Medicine, University of Campania “Luigi Vanvitelli”, 80100 Naples, Italy; annalisa.capuano@unicampania.it

**Keywords:** PD-L1, microvesicles, liquid biopsy, chemotherapy, immunotherapy, pancreatic cancer

## Abstract

**Simple Summary:**

There is an urgent need to improve the therapeutic options in pancreatic cancer. In this study, we assessed the ability of two standard-of-care chemotherapeutic regimens to modulate the levels of PD-L1 mRNA isolated from plasma-derived microvesicles (MVs) of patients with pancreatic cancer. Our findings demonstrate for the first time a statistically significant difference in MV-derived PD-L1 mRNA levels at 3 months vs. baseline in patients treated with GEMnPAC, compared to those receiving FOLFIRINOX. Although these findings need to be confirmed in larger prospective cohorts, they can represent a rational basis for testing the hypothesis that the GEMnPAC schedule may be used as an immunotherapy-modulating regimen in PDAC patients due to its capability of increasing PD-L1 mRNA expression.

**Abstract:**

Pancreatic ductal adenocarcinoma (PDAC) is a non-immunogenic tumor poorly responsive to immune checkpoint inhibitors. This study investigates the effect of 5-fluorouracil (5-FU), irinotecan, and oxaliplatin (FOLFIRINOX), and gemcitabine plus nab-paclitaxel (GEMnPAC) regimens on PD-L1 mRNA expression in plasma-derived microvesicles (MVs) in 50 PDAC patients. Plasma was collected before starting chemotherapy and after 3 months of treatment. mRNA was extracted from MVs, and PD-L1 expression was measured by digital droplet PCR. Twenty-eight patients were PD-L1 positive in MVs at baseline, of which 18 were in the GEMnPAC cohort and 10 in the FOLFIRINOX one. The amount of PD-L1 expression in MVs increased from baseline to 3 months of treatment in patients receiving GEMnPAC (median value 0.002 vs. 0.005; *p* = 0.01) compared to those treated with FOLFIRINOX (median 0.003 vs. 0.004; *p* = 0.97). The increase in PD-L1 mRNA expression in MVs was not related to tumor response (PR + SD: *p* = 0.08; PD: *p* = 0.28). Our findings demonstrate that GEMnPAC can increase PD-L1 mRNA expression in patient-derived circulating MVs, providing a rationale for testing the efficacy of this regimen in sequential or simultaneous combinations with immunotherapy in PDAC patients.

## 1. Introduction

Pancreatic ductal adenocarcinoma (PDAC) represents one of the leading causes of global cancer deaths in industrialized countries [1]. The overall 5-year survival for patients affected by the locally advanced or metastatic disease showed minimal improvements during recent years, with a rate less than 6% [1] and, despite the application of advanced treatment approaches, PDAC therapy remains a formidable challenge. The combination of 5-fluorouracil (5-FU), irinotecan, and oxaliplatin (FOLFIRINOX), and of gemcitabine and nab-paclitaxel (GEMnPAC), showed an improvement in survival of PDAC patients and represents the standard of care as first-line treatment [2]. However, recurrence is nearly inevitable for most PDAC patients and the development of chemo-resistance is the main factor limiting treatment efficacy [3].

The immune checkpoint programmed death 1 (PD-1) receptor and its ligand PD-L1 are expressed on the surface of activated T lymphocytes, dendritic cells and macrophages, and are crucial for the immune system, acting as co-inhibitory regulators and limiting the development of T cell response, thus reducing the risk of autoimmunity [4]. It has now been recognized that PD-L1 expression is a common feature of several tumor types and its overexpression may result in an aggressive phenotype with a poor prognosis [5,6,7,8]. Recently, inhibition of the PD-1/PD-L1 axis by immune checkpoint inhibitors (ICIs) has substantially changed the therapeutic landscape of several malignancies, including melanoma [9], non-small cell lung cancer [10,11], urothelial carcinoma [12], and renal cancer [13].

PD-L1 is also expressed in PDAC [14,15], providing the rationale of exploring immunotherapy agents in this setting. Despite initial encouraging results in preclinical models, ICIs monotherapy failed in several clinical studies, showing no objective responses [16,17]. Consistent with other tumors, PD-L1 expression has a negative prognostic role, as it is associated with poorer survival in subjects affected by PD-L1 positive PDAC (one-year post-operative survival rate: 33.5% vs. 60.3%; *p* = 0.016) [18]. Interestingly, promising activity of gemcitabine and anti-PD-L1 combination has been shown in a preclinical study, providing evidence of a synergy between these drugs [18]. Starting from this observation, the present study was aimed at investigating the effect of FOLFIRINOX and GEMnPAC on PD-L1 mRNA expression in PDAC patients, using plasma-derived microvesicles (MVs) as a tool to evaluate tumor dynamics.

## 2. Patients and Methods

### 2.1. Patient Selection

This is a mono-institutional retrospective translational study carried out on PDAC patients treated with first-line FOLFIRINOX or GEMnPAC. Patients were accrued from March 2017 to September 2019 at the Medical Oncology Unit of the University Hospital of Pisa (Italy). Inclusion criteria were: histologically proven PDAC and age ≥ 18 years-old, diagnosis of metastatic or locally advanced PDAC not amenable by surgical resection, clinical data and blood samples available for analysis. The chemotherapy regimen was at the investigator’s choice. Complete or partial responses (CR, PR), disease stabilization (SD), and disease progression (PD) were defined by imaging, according to RECIST 1.1 criteria. Blood samples were obtained for the analysis of MV-derived PD-L1 mRNA at baseline and after 3 months of treatment, i.e., at the first radiological assessment of disease status. Approval was obtained by the local Ethics Committee (code n. 12159); the study was carried out in line with the Declaration of Helsinki, and signed informed consent was obtained from all participants.

### 2.2. MVs Isolation and Assessment of PD-L1 mRNA Expression

Blood samples (12 mL) were obtained at baseline and after 3 months of treatment and MVs and mRNA were isolated using the exoRNeasy kit (Qiagen, Valencia, CA, USA). The expression levels of PD-L1 mRNA were measured using a QX100 ddPCR (Bio-Rad, Hercules, CA, USA), the One-Step RT ddPCR kit, and the PrimePCR ddPCR Expression Probe Assay for CD274 (human). The human β-actin ddPCR assay (Bio-Rad) was used as a reference gene and internal control. mRNA expression levels were assessed by the automatic quantification of signals generated by the droplet reader using the QuantaSoft software (Bio-Rad). Data are reported as % fractional abundance (FA).

### 2.3. Statistical Analysis

Variables including gender, tumor stage, Eastern Cooperative Oncology group performance status (ECOG PS), primary tumor location, liver and lung metastases were reported as absolute and relative frequencies. Baseline values, such as age, CA 19-9 (carbohydrate antigen 19-9), NLR (neutrophil-lymphocyte ratio), PLR (platelet-lymphocyte ratio) were dichotomized via a median split and treated as categorical variables. The number of metastatic sites was dichotomized in two groups (1–2 vs. 3–5). The MVs PD-L1 mRNA expression was assessed both as categorical (negative—when the value was 0, vs. positive—when the value was > 0; and increase vs. no increase) and continuous variables. The Kolmogorov–Smirnov test was used to assess the normality of the quantitative data distribution. To compare categorical variables with PD-L1 mRNA expression, the Mann–Whitney test (two-tailed) was carried out, while the Wilcoxon test (two-tailed) was used to evaluate the paired data (matched). The effect size measure was calculated using Cohen’s d. Radiological progression-free survival (PFS) and overall survival (OS) were measured from the date of the first cycle of chemotherapy to disease progression/death and to death/last follow-up visit, respectively. PFS and OS curves were estimated using the Kaplan–Meier method and a log-rank test was used to evaluate differences between curves. Univariate analysis was performed to correlate PFS and OS with demographics, clinical status of patients and laboratory parameters, i.e., age, gender, tumor stage, ECOG PS, primary tumor location, baseline CA 19-9, NLR and PLR, number of metastatic sites, chemotherapy regimen and PD-L1 mRNA expression. Multivariate analysis was performed by Cox proportional hazards regression modeling and the results were expressed by hazard ratios (HR) with confidence interval 95% (CI) and related p-value. Differences were considered statistically significant at *p* < 0.05. Statistical analyses, descriptive and inferential, were carried out by SigmaPlot version 12 (Systat Software, San Jose, CA, USA), and GraphPad Prism version 5 (GraphPad Software, La Jolla, CA, USA).

## 3. Results

### 3.1. Patient Characteristics

Fifty patients with locally advanced (*n* = 16; 32%) and metastatic (*n* = 34; 68%) PDAC were included in this study. Thirty-four patients (68%) were treated with GEMnPAC (2 locally advanced and 32 metastatic), while 16 patients (32%) received FOLFIRINOX (14 locally advanced and 2 metastatic). Median age was 60.5 years (range 33–76). At the first radiological assessment, 9 patients (26.5%) given GEMnPAC obtained a PR, 16 (47%) had a SD and 9 (26.5%) progressed. In the FOLFIRINOX group, there were 2 (12.5%) PRs, 10 (62.5%) SDs and 4 (25%) PDs. Table 1 describes the clinical features of patients, and response to chemotherapy. Overall, median PFS and OS were 6.6 and 8.6 months, respectively. Overall response rate (ORR) was 22% and disease control rate (DCR), calculated as the sum of PR and SD, was 74%.

### 3.2. PD-L1 mRNA Expression in MVs

In 22 out of 50 patients (44%), PD-L1 mRNA expression in MVs was undetectable at baseline, while 28 of them (56%) were PD-L1-positive (median FA 0.005 %; range 0.0007–0.0183%). Among PD-L1-expressing patients, 18 received GEMnPAC and 10 FOLFIRINOX. After 3 months of treatment with GEMnPAC and FOLFIRINOX, 26 and 12 patients resulted positive, respectively. Only 1 patient was not evaluable for PD-L1 mRNA expression. In the GEMnPAC cohort, levels of PD-L1 mRNA increased in 18 patients and were stable/decreased in 15 patients. At variance with this, PD-L1 mRNA expression was increased in 5 patients treated with FOLFIRINOX, and remained stable/decreased in 11 patients (Table 1 and Table 2).

**Figure 1:**
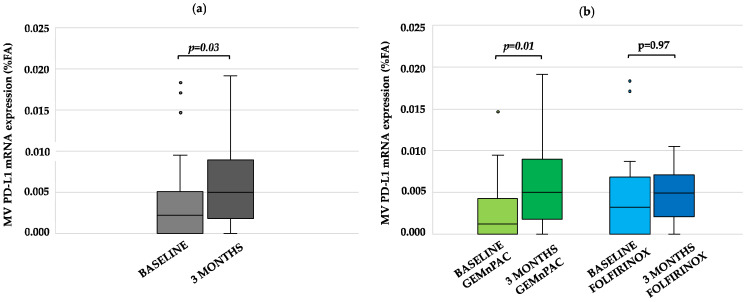
Effect of chemotherapy on PD-L1 mRNA levels in pancreatic cancer. (**a**) PD-L1 mRNA expression evaluated at baseline (light grey) and at tumor re-assessment after 3 months of treatment (dark grey), in the overall population; (**b**) differences in PD-L1 mRNA expression between baseline (light green and blue) vs. 3 months (dark green and blue) in patients grouped according to chemotherapy regimen (GEMnPAC vs. FOLFIRINOX). *p* values are reported on top of the bar chart. Abbreviations: PD-L1, programmed death-ligand 1; FA, fractional abundance.

Overall, a significant increase in MV-derived PD-L1 mRNA levels was observed at 3 months of treatment compared to baseline (median FA 0.0022% vs. 0.0049%; *p* = 0.033; Cohen’s d = 0.5). In particular, stratifying patients according to treatment arms, PD-L1 mRNA levels were higher in patients receiving GEMnPAC than in those treated with FOLFIRINOX (GEMnPAC: median FA 0.002 vs. 0.005; *p* = 0.01; Cohen’s d = 0.7; FOLFIRINOX: median FA 0.003% vs. 0.004%; *p* = 0.97). The bar chart depicting the difference in PD-L1 mRNA expression at baseline vs. 3 months, in the whole population and in the two cohorts of patients, is shown in Figure 1a,b respectively.

**Figure 2:**
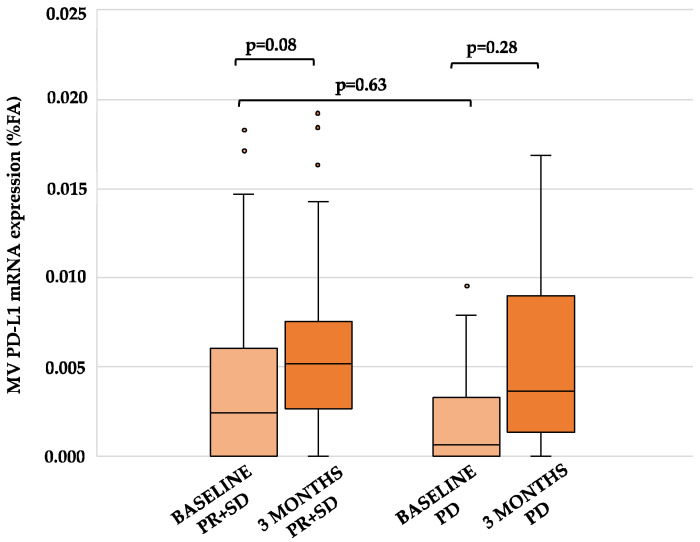
Effect of chemotherapy on PD-L1 mRNA levels and clinical response in PDAC patients. The amount of PD-L1 mRNA (FA) at baseline (light orange) and at tumor re-assessment after 3 months of treatment (dark orange) was grouped according to tumor response (PR + SD and PD); p values are reported on top of the bar chart. Abbreviations: PD-L1, programmed death-ligand 1; FA, fractional abundance; PR, partial response; SD, stable disease; PD, progressive disease.

PD-L1 dynamics were not related to treatment response, since the expression of PD-L1 at baseline vs. 3 months of treatments was not significantly modified in patients who had a disease control (PR + SD; *p* = 0.08), compared to those who progressed (*p* = 0.28). Moreover, no statistically significant difference in baseline PD-L1 mRNA expression between patients who achieved PR + SD and PD was found (*p* = 0.63; Figure 2).

No statistical differences in PD-L1 mRNA levels were observed at baseline when patients were stratified by different clinico-pathological and laboratory features, such as age, gender, stage, ECOG PS, primary tumor location, baseline CA 19-9, NLR and PLR levels, as well as the number of metastatic sites and presence/absence of liver or lung metastases (all *p* > 0.05; Table 3).

**Figure 3:**
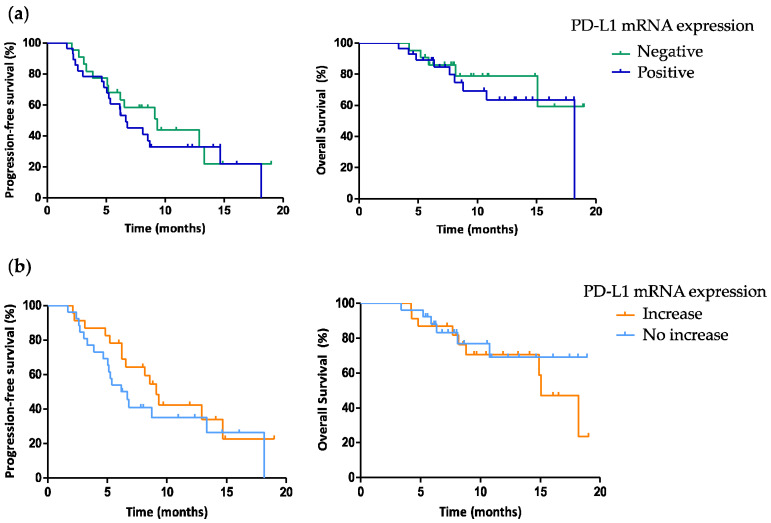
(**a**) Progression-free survival and overall survival in patients with or without detectable PD-L1 at baseline and (**b**) with increase vs. no increase in PD-L1 mRNA expression. Abbreviation: PD-L1, programmed death-ligand 1.

No differences in PFS (HR 1.321, 95% CI 0.657–2.660, *p* = 0.435) and OS (HR 1.529, 95% CI 0.508–4.604, *p* = 0.45) were reported in patients not expressing PD-L1 (*n* = 22) with respect to those PD-L1-positive at baseline (*n* = 28). Similarly, no significant differences in PFS (HR 0.672, 95% CI 0.333–1.357, *p* = 0.268) and OS (HR 1.280, 95% CI 0.441–3.713, *p* = 0.65) were observed in patients with increase (*n* = 23) vs. no increase (*n* = 26) of PD-L1 mRNA levels (Figure 3a,b).

## 4. Discussion

Chemotherapy represents the standard of care in PDAC patients with advanced disease. However, chemoresistance remains the main factor affecting treatment outcome. Therefore, understanding biological changes associated with the resistant phenotype may be helpful to instruct a more effective treatment. Recently, the discovery of PD-(L)1 as one of the most successful druggable targets for immune regulation, has generated great interest, leading ICIs being tested in several clinical trials for a wide range of solid tumors [9,10,11]. It has been reported that PD-L1 is a dynamic biomarker that can be induced by chemotherapy or radiotherapy [19,20]. Therefore, this study investigated the effect of chemotherapy (i.e., GEMnPAC and FOLFIRINOX) on the expression levels of PD-L1 mRNA isolated from plasma-derived MVs in PDAC patients.

The positive rates of PD-L1 protein expression in PDAC are controversial in literature, as they are highly heterogenous, ranging from 9% to 62.5% [18,21,22,23,24,25]. Furthermore, the positive rates further change when PD-L1 mRNA expression is analyzed, with a range between 19% to 50% [26,27]. This marked difference may be mainly attributed to the different technique used for PD-L1 detection, including antibodies for immunohistochemistry staining and the staining scoring criteria, as well as to the effect of previous treatments. Furthermore, the assessment of PD-L1 expression in primary tumors at diagnosis may not provide information on changes associated with clonal evolution and specific pharmacological pressure [28]. Thus, a growing number of studies have shifted the attention on circulating biomarkers, since they can provide meaningful information on tumor dynamics and can be reliable tools to monitor treatment response and clinical outcome [29,30]. More specifically, the use of MVs in this study was suggested by their ability to preserve intact mRNA and their role in cell–cell interaction [30]. Indeed, we were able to show that 56% of PDAC patients had detectable PD-L1 mRNA levels prior to treatment.

Although patients with PDAC may show detectable PD-L1 mRNA levels, no promising results have been obtained with ICIs monotherapy thus far [15,31].

Interestingly, several studies reported the ability of conventional chemotherapy or radiotherapy to induce PD-L1 expression. Ng and collaborators demonstrated that in vitro and in vivo treatments with carboplatin plus paclitaxel or 5-FU plus cisplatin could induce PD-L1 expression in esophageal squamous cell carcinoma cells [32]. Similarly, treatment with gemcitabine, paclitaxel and 5-FU and radiotherapy has been reported to cause an induction of PD-L1 protein expression in pancreatic cancer cells [19,20]. Although preclinical investigations have already demonstrated that PD-L1 expression is differentially regulated by chemotherapy, it remains to be further elucidated whether this mechanism can be translated to PDAC patients. To address this point, we explored the change in PD-L1 mRNA expression in plasma-derived MVs of PDAC patients treated with FOLFIRINOX or GEMnPAC. The results showed, for the first time, a statistically significant difference in PD-L1 mRNA levels from baseline to 3 months after starting treatment in patients given GEMnPAC, compared to those receiving FOLFIRINOX. As already reported, over-expression of PD-L1 may reduce the sensitivity of cancer cells to chemotherapy, favoring the escape from immune surveillance and leading to the development of chemoresistance [33]. Therefore, if chemotherapy induces PD-L1 expression, rendering cancer cells more susceptible to ICIs, the use of standard chemotherapy in combination with ICIs may be more beneficial than ICI monotherapy.

The increase in PD-L1 expression has been also investigated in this study as a putative predictive biomarker of resistance to chemotherapy. Unfortunately, the PFS and OS did not show statistically significant differences, even if a difference in terms of PFS was seen. However, since this analysis may be biased by the small number of samples analyzed, further investigation in a larger cohort of patients may demonstrate that this non-invasive approach could be used to determine which patients might benefit from a combined treatment of chemotherapy and ICIs.

The safety and tolerability of chemotherapy (gemcitabine +/− nab-paclitaxel) in combination with ICIs (tremelimumab, ipilimumab, pembrolizumab or nivolumab) have been preliminarily explored in metastatic PDAC patients. Along with a manageable safety profile, interesting results have been reported in terms of PFS and OS, despite being preliminary. It is of note that tremelimumab/gemcitabine combination granted a median OS of 7.4 months and 2 PR were achieved among the 34 patients treated [34]; similar results were described for 21 advanced PDAC patients treated with ipilimumab/gemcitabine combination (median OS: 6.9 months; ORR; 14%) [35]. Even more interest was raised by the results of the combination of GEMnPAC and anti-PD-(L)1 agents. Indeed, despite its primary endpoint of >15%, complete response was not met; GEMnPAC plus pembrolizumab achieved 9.0 and 15.0 months of median PFS and OS, respectively, among 17 patients enrolled in single center phase Ib/II trial [36]. Consistently, 64% of disease control rate (DCR) and 18% of overall response rate (ORR) were recently reported with GEMnPAC and nivolumab triplet [37].

Unfortunately, the above-mentioned signals of chemo/immune synergy were not confirmed by the recent phase II randomized PA.7 trial, which compared the doublet standard chemotherapy with the addition of durvalumab and tremelimumab; no significant differences were seen neither in OS nor in PFS, in spite of a slightly non-significantly higher efficacy of the experimental arm [38].

This failure emphasizes the need for predictive biomarkers of response to ICI-based regimens for advanced PDAC patients. At present, the most investigated one is PD-L1, the expression of which changes in response to chemotherapy in solid tumors such as in urothelial carcinoma treated with cisplatin/carboplatin and paclitaxel or in human adenocarcinoma and hepatocellular cell lines [39,40].

Recently, a meta-analysis suggested that high PD-L1 expression levels can be associated with clinicopathologic characteristics including advanced tumor stage and low differentiation [41]. However, our study showed that PD-L1 mRNA expression in MVs at baseline was not correlated with any clinicopathological feature or laboratory parameter, including CA 19-9, NLR and PLR. Finally, the role of PD-L1 as a prognostic biomarker has not been yet fully elucidated in pancreatic cancer, mainly due to its non-immunogenic nature, and published data still remain controversial [14,42,43].

Concerning the mechanisms by which cytotoxic drugs may modulate the expression of PD-L1 in this patient population, only assumptions can be made. It has been reported that the JAK2/STAT1 pathway may be involved in the regulation of PD-L1 expression in pancreatic cancer cells, which is increased by gemcitabine or paclitaxel [19]. Irinotecan upregulates PD-L1 expression on tumor cells [44], while oxaliplatin and 5-FU downregulate PD-L1 in colon cancer cells [45]. The opposing effects on PD-L1 expression induced by the single agents of the FOLFIRINOX combination may therefore account for findings of the current study showing no change in PD-L1 mRNA levels in PDAC patients treated with this schedule.

In agreement with the previous data, a recent work demonstrated that the combination of anti-PD-1 ICI with gemcitabine is active in the treatment of a murine model of PDAC liver metastasis and is capable of enhancing the immune response mediated by Th1 lymphocytes and M1 macrophages and was associated with recruitment of CD8+ T cells [46].

To better define the origin of MVs and the cargo they transport, we considered that circulating mutant KRAS DNA varies according to tumor response [47], unlike PD-L1 observed in this study, which does not parallel tumor changes, thus ruling out that the increase in PD-L1 in plasma is dependent on the release of biological macromolecules by dying cells as a result of chemotherapy.

This study has limitations due to its retrospective nature and inclusion of a relatively small number of patients. However, it is founded on the evidence that PD-L1 expression is detected in pancreatic carcinoma [48] and the PD-L1 protein is commonly found on the surface of tumor-derived MVs [49] including PDAC [50]. Therefore, it is conceivable that this may have also been the case with the PDAC patients included in our study.

## 5. Conclusions

Our findings support the hypothesis that GEMnPAC schedule may be used as an induction-treatment in PDAC patients treated with ICIs due to its capability to increase PD-L1 mRNA expression. Although data need to be confirmed in larger prospective cohorts, they can represent a rational basis for testing sequential or simultaneous chemotherapy-immunotherapy combinations in PDAC patients.

## Figures and Tables

**Table 1:** Clinical characteristics of patients.

		N	%
Gender	Male	25	50
	Female	25	50
Age	Median (range)	60.5 (33–76) years	
ECOG PS	0	34	68
	1	16	32
Chemotherapy	FOLFIRINOX	16	32
	GEMnPAC	34	68
Stage	III	16	32
	IV	34	68
Primary tumor location	Head	23	46
	Body-tail	27	54
Number of metastatic sites	1–2	36	72
	3–5	14	28
Liver metastases	No	16	32
	Yes	21	42
	NA	13	26
Lung metastases	No	28	56
	Yes	9	18
	NA	13	26
Baseline CA 19-9 (U/mL)	median (range)	266.6 (0.1–120,000)	
	≤median	25	50
	>median	24	48
	NA	1	2
Baseline NLR	median (range)	2.21 (0.538–9.46)	
	≤median	24	48
	>median	23	46
	NA	3	6
Baseline PLR	median (range)	128.99 (60.76–409.9)	
	≤median	24	48
	>median	23	46
	NA	3	6
PD-L1 mRNA at baseline (%FA)	Negative	22	44
	Positive	28	56
PD-L1 mRNA at 3 months (%FA)	Negative	11	22
	Positive	38	76
	NA	1	2

Abbreviations: N, number; ECOG PS, Eastern Cooperative Oncology Group performance status; GEMnPAC, gemcitabine plus nab-paclitaxel; FOLFIRINOX, 5-FU, irinotecan, and oxaliplatin; CA 19-9, carbohydrate antigen 19-9; NA, not available; NLR, neutrophil-lymphocyte ratio; PLR, platelet-lymphocyte ratio; PD-L1, programmed death-ligand 1; FA, fractional abundance.

**Table 2:** PD-L1 fractional abundance (FA) in plasma-derived MVs at baseline and at first tumor assessment (3 months) vs. tumor response in patients treated with GEMnPAC or FOLFIRINOX.

GEMnPAC Group					FOLFIRINOX Group				
Patient	PD-L1 FA (Baseline)	PD-L1 FA (3 Months)	PD-L1 FA Change	Tumor Response	Patient	PD-L1 FA (Baseline)	PD-L1 FA (3 Months)	PD-L1 FA Change	Tumor Response
1	0.007	0.005	↓	PR	35	0.007	0.004	↓	SD
2	0	0.005	↑	SD	36	0.018	0.007	↓	PR
3	0	0	=	PR	37	0	0	=	PD
4	0.007	0.014	↑	SD	38	0	0.007	↑	PD
5	0.004	0.007	↑	SD	39	0.001	0	↓	SD
6	0.009	0.009	=	PD	40	0	0.009	↑	SD
7	0.002	0.012	↑	PR	41	0	0	=	SD
8	0	0.002	↑	PD	42	0	0	=	SD
9	0.005	0.003	↓	PD	43	0.009	0.003	↓	PR
10	0	0.017	↑	PD	44	0	0.009	↑	PD
11	0.002	0.008	↑	SD	45	0.007	0.006	↓	SD
12	0.003	0.005	↑	SD	46	0.017	0.006	↓	SD
13	0.005	0.003	↓	SD	47	0.006	0.01	↑	SD
14	0.015	0.003	↓	SD	48	0.005	0.006	↑	SD
15	0.004	0	↓	SD	49	0.003	0.003	=	PD
16	0.008	0.004	↓	PD	50	0.004	0.004	=	SD
17	0	0	=	PR					
18	0.008	0.006	↓	SD					
19	0	0.018	↑	SD					
20	0	0	=	PD					
21	0	0.016	↑	PR					
22	0.001	0	↓	PD					
23	0	0.004	↑	PR					
24	0	0.009	↑	SD					
25	0	0.019	↑	SD					
26	0	0	=	SD					
27	0	0.011	↑	PR					
28	0	0.003	↑	PR					
29	0.003	0.001	↓	PR					
30	0.002	0.015	↑	PD					
31	0	0	=	SD					
32	0	0.005	↑	SD					
33	0.004	0.006	↑	SD					
34	0.005	NE	NE	PD					

Abbreviations: ↑, increase; ↓, decrease; =, no variation; NE, not evaluable.

**Table 3:** Correlation of PD-L1 mRNA levels at baseline with clinical status of patients and laboratory parameters.

		PD-L1 mRNA FA (Median, %)	*p*-Value
Age	<60.5 vs. ≥60.5	0.00125 vs. 0.00243	0.83
Gender	Male vs. Female	0 vs. 0.00272	0.43
Stage	III vs. IV	0.00505 vs. 0.00347	0.06
ECOG PS	0 vs. 1	0.00257 vs. 0.0011	0.44
Primary tumor location	Body-tail vs. Head	0 vs. 0.00367	0.14
Baseline CA 19-9	≤median 266.6 vs. >median 266.6	0.00359 vs. 0	0.29
Baseline NLR	≤median 2.21 vs. >median 2.21	0.0031 vs. 0.000693	0.39
Baseline PLR	≤median 128.99 vs. >median 128.99	0.00173 vs. 0.00243	0.44
Number of metastatic sites	1–2 vs. 3–5	0.000973 vs. 0.003	0.43
Liver metastases	Yes vs. No	0.000693 vs. 0.00287	0.38
Lung metastases	Yes vs. No	0 vs. 0.00173	0.91

Abbreviations: FA: fractional abundance; ECOG PS: Eastern Cooperative Oncology Group performance status; CA 19-9: carbohydrate antigen 19-9; NLR: neutrophil-lymphocyte ratio; PLR: platelet-lymphocyte ratio.

## Data Availability

The data presented in this study are available in this article.
